# Chiral perturbation theory for neutron–antineutron oscillations

**DOI:** 10.1140/epjc/s10052-017-5411-7

**Published:** 2017-12-14

**Authors:** Johan Bijnens, Erik Kofoed

**Affiliations:** 1 Department of Astronomy and Theoretical Physics, Lund University, Sölvegatan 14A, 223-62 Lund, Sweden; 2 Narodowe Centrum Badań Ja̧drowych, Hoża 69, 00-681 Warsaw, Poland

## Abstract

We construct the Chiral Perturbation Theory operators for neutron–antineutron oscillations and use these to estimate chiral and finite volume corrections at one-loop order.

## Introduction

The baryon asymmetry of the universe is one of the open problems in particle physics. One possible solution is to have $B-L$ violation as exemplified in $\Delta B=2$ transitions and in particular neutron–antineutron oscillations. This has been suggested long ago; see e.g. [1–5]. Recent reviews are [6, 7]. $\Delta B=2$ transitions require a six-quark operator. These were classified in [8–10]. To obtain predictions of a particular model the coefficients of these operators need to be evolved to a low scale and then the matrix elements computed. This running is known to two-loop order [11]. We will also use the notation of the operators used in that reference. In the past these matrix elements were estimated using models but now the first lattice calculations have appeared [12, 13]. These can be done at different quark masses from the physical ones and are necessarily at finite volume. Chiral Perturbation Theory (ChPT) allows to do estimate both of these effects.

The bounds on the mean oscillation time $\tau$ are $\tau > 8.6 \times 10^{7}$ s from free neutrons [14] and $\tau > 2.7 \times 10^{8}$ s from bound neutrons [15]. The reason why the bound from bound neutrons is much lower than those for proton decay is that the antineutron inside nuclei is far off-shell, see e.g. [16] for a clear explanation. For the same reason, strong magnetic shielding is needed for the free neutron experiments. A new free neutron experiment is proposed for ESS in Lund [17] so a better estimate of the matrix elements will be very useful to put limits on $\Delta B=2$ effects in theories beyond the Standard Model.

In this paper we construct the ChPT equivalents of the six-quark operators of [11] and use these then to calculate the chiral and finite volume corrections in the isospin limit. The finite volume corrections are found to be small for $m_\pi L>4$ for the physical pion mass but chiral extrapolations can be substantial already for pion masses of order 200 MeV.

In Sect. 2 we discuss shortly the quark operators of [11] and their chiral representation. Sect. 3 discusses the ChPT aspects. The main new result is the construction of the ChPT operators for neutron–antineutron transitions. This is done using the spurion technique. In Sect. 4 we calculate the one-loop corrections in ChPT to the matrix elements and in Sect. 5 we give some numerical results. Our main conclusions are given in Sect. 6. Appendix A recalls some *SU*(2) identities used heavily in deriving the ChPT operators and the needed integrals are discussed in Appendix B.

Preliminary results of this work were presented in the master thesis [18] and at Lattice 2017 [19]. Related work is in progress by Oosterhof et al. [20].

## Quark operators and chiral properties

The operator structure needed for $n\bar{n}$-transitions contains six quark fields *dddduu* where under the chiral symmetry group $SU(2)_\mathrm{L}\times SU(2)_\mathrm{R}$ each quark field can be in a left- or right-handed doublet. The operators were classified in [8–10] and rewritten in a basis that shows the chiral properties in [11]. It was found that there are 14 operators that have six types of representations under the chiral group. There are three $(1_\mathrm{L},3_\mathrm{R})$, one $(1_\mathrm{L},7_\mathrm{R})$ and three $(5_\mathrm{L},3_\mathrm{R})$ operators, as well as their parity conjugates. The chiral loop corrections for the parity-conjugates are the same since the strong interactions are invariant under parity.

If we assume isospin conservation, only an $I=1$ operator can contribute to $n\bar{n}$-transitions. So only the $I=1$ projection of the different $(5_\mathrm{L},3_\mathrm{R})$ and $(3_\mathrm{L},5_\mathrm{R})$ operators contributes, this explains why the loop contributions for all those operators are the same, in fact one can show that the operators $P_5,P_6,P_7$ (and similarly $Q_5,Q_6,Q_7$) are related by isospin. The $(1_\mathrm{L},7_\mathrm{R})$ and $(7_\mathrm{L},1_\mathrm{R})$ operators do not contribute in the isospin limit. The operators are summarized in Table 1.

**Table 1:** The chiral representations of the dimension-9 six-quark operators as listed in [11] as well as the corresponding spurions. The indices on the spurions are $SU(2)_\mathrm{L}\times SU(2)_\mathrm{R}$ upper doublet, fully symmetrized in the indices of the same type

Chiral	$\#$Operators	Chiral	Spurion	$\#$Operators
$(3_\mathrm{L},1_\mathrm{R})$	3: $P_1,P_2,P_3$	$\theta _i^{i_\mathrm{L} j_\mathrm{L}}~(i=1,2,3)$	$(1_\mathrm{L},3_\mathrm{R})$	3: $Q_1,Q_2,Q_3$
$(3_\mathrm{L,}5_\mathrm{R})$	3: $P_5,P_6,P_7$	$\theta _i^{i_\mathrm{L} j_\mathrm{L} k_\mathrm{R} l_\mathrm{R} m_\mathrm{R} n_\mathrm{R}}~(i=4,5,6)$	$(3_\mathrm{R},5_\mathrm{L})$	3: $Q_5,Q_6,Q_7$
$(7_\mathrm{L},1_\mathrm{R})$	1: $P_4$	$\theta _4^{i_\mathrm{L} j_\mathrm{L} k_\mathrm{L} l_\mathrm{L} m_\mathrm{L} n_\mathrm{L}}$	$(1_\mathrm{L},7_\mathrm{R})$	1: $Q_4$

We can add spurion fields transforming under $G_\chi =SU(2)_\mathrm{L}\times SU(2)_\mathrm{R}$ such that the combination of quark-operators with chiral flavour indices and the spurions is invariant under $G_\chi$. These will be used to construct the operators in ChPT. There is a corresponding set for the opposite parity operators $Q_i$.

## Chiral perturbation theory

We work in two-flavour ChPT and we use the heavy-baryon formalism [21] (HBCHPT), a review and introduction is [22]. The notation we use can be found in [22] or [23]. The lowest order meson Lagrangian is

1 $$\begin{aligned} \mathcal {L}_2 =\,&\frac{F^2}{4}\left\langle u_\mu u^\mu +\chi _+\right\rangle ,\quad u_\mu = i\left[ u^\dagger (\partial _\mu -i r_\mu )u\right. \nonumber \\&~~~~~~~~~~~~~~~~~~~~~~~~~~~~~~~~~~~~~~~~~~~\left. -u(\partial _\mu -i l_\mu )u^\dagger \right] , \nonumber \\ \chi =\,&2 B\left( s+i p\right) , \quad \Gamma _\mu =\, \frac{1}{2}\left[ u^\dagger (\partial _\mu -i r_\mu )u\right. \nonumber \\&~~~~~~~~~~~~~~~~~~~~~~~~~~~~~~~~~~~~~~~~\left. -u(\partial _\mu -i l_\mu )u^\dagger \right] , \nonumber \\ \chi _\pm =\,&u^\dagger \chi u^\dagger \pm u \chi ^\dagger u,\quad \left\langle A \right\rangle \equiv \, \mathrm {tr}(A). \end{aligned}$$

*u* is a $2\times 2$ unitary matrix that contains the pion fields $\pi ^a$ via $u=\exp (\pi ^a \tau ^a/(2F))$, with $\tau ^a$ the Pauli matrices. *B*, *F* are the two lowest-order (LO) low-energy constants (LECs). The $2 \times 2$ matrices $s,p,l_\mu ,r_\mu$ are the usual ChPT external fields.

Under a chiral transformation $g_\mathrm{L},g_\mathrm{R}$ the objects above transform as

2 $$\begin{aligned} u\rightarrow \,&g_\mathrm{L} u h^\dagger \equiv h u g_\mathrm{L}^\dagger ,&u_\mu \rightarrow \,&h u_\mu h^\dagger ,&\chi \rightarrow \,&g_\mathrm{R}\chi g_\mathrm{L}^\dagger , \nonumber \\ \chi _\pm \rightarrow \,&h\chi _\pm h^\dagger ,&U=u^2\rightarrow \,&g_\mathrm{R} U g_\mathrm{L}^\dagger . \end{aligned}$$

The first equation is the definition of the compensator transformation *h* which depends on $u,g_\mathrm{L},g_\mathrm{R}$. The last one defines *U*.

Nucleons in a relativistic normalization can be included via a doublet field $\Psi$ at LO as [24]

3 $$\begin{aligned} \Psi =\,&\left( \begin{array}{c}p\\ n \end{array} \right) ,&\psi \rightarrow \,&h \Psi , \nonumber \\ \mathcal {L}_\mathrm{R} =\,&\overline{\Psi }\left( iD_\mu \gamma ^\mu - m+\frac{g_A}{2}u_\mu \gamma ^\mu \gamma _5\right) \Psi ,&D_\mu \equiv \,&\partial _\mu +\Gamma _\mu . \end{aligned}$$

In HBCHPT we project on velocity-dependent fields $\mathcal {N}$ via

4 $$\begin{aligned} \mathcal {N}=(1/2)(1+v_\mu \gamma ^\mu )\exp (i mv\cdot x)\Psi , \end{aligned}$$

with *v* a four-velocity with $v^2=1$. However, in this paper we need to introduce also an antinucleon field with the same velocity *v*. The charge conjugate fermion spinor is $\psi ^c\equiv -i\gamma ^2\psi ^*$. We then define5The transformation under the chiral group follows from the properties of *SU*(2) using the identities in Appendix A. We then define a HBCHPT field for the antineutron as

6 $$\begin{aligned} \mathcal {N}^c=(1/2)(1+v_\mu \gamma ^\mu )\exp (i mv\cdot x)\Psi ^c. \end{aligned}$$

**Fig. 1:**
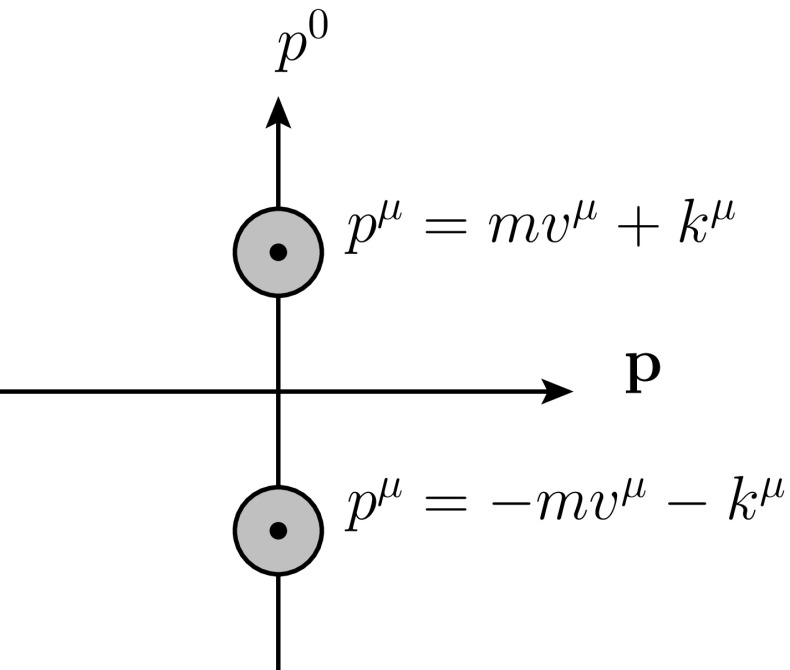
A pictorial representation of the velocity regions relevant for projection on the nucleon and antinucleon HBCHPT fields

Compared to the first projection (4), this is at $-v$ if formulated in terms of $\Psi$. $\mathcal {N}$ and $\mathcal {N}^c$ are in HBCHPT independent fields, since they are from expansions around different widely-separated velocities as depicted in Fig. 1. The lowest order Lagrangian for the HBCHPT fields is

7 $$\begin{aligned}&\mathcal {L}_{N} =\overline{\mathcal {N}}\left( i v^\mu D_\mu + g_A u^\mu S_\mu \right) \mathcal {N}\nonumber \\&~~~~~~\quad +\overline{\mathcal {N}^c}\left( i v^\mu D_\mu - g_A u^\mu S_\mu \right) \mathcal {N}^c. \end{aligned}$$

The signs can be derived using charge conjugation. The spin vector $S_\mu$ has the properties

8 $$\begin{aligned} S_\mu =\,&-\frac{1}{4}\gamma _5\left[ \gamma _\mu ,\gamma _\nu \right] v^\nu ,&S^2 =\,&\frac{1-d}{4},&\left\{ S_\mu ,S_\nu \right\} \nonumber \\&= \frac{1}{2}\left( v_\mu v_\nu -g_{\mu \nu }\right) ,&v\cdot S =\,&0. \end{aligned}$$

These properties are sufficient for our calculation. Higher order Lagrangians can be constructed in the same way as usual.

The operators that give neutron–antineutron transitions have to be written with doublet indices and must create the antineutron. For this we introduce^1^

9 $$\begin{aligned} \widetilde{\mathcal {N}^c}=\,&\left( \begin{array}{c}\overline{p^c}\\ \overline{n^c}\end{array}\right) = -i\tau ^2{\overline{\mathcal {N}^c}}^T,&\widetilde{\mathcal {N}^c}\rightarrow \,&h\widetilde{\mathcal {N}^c}. \end{aligned}$$

We need to construct operators that transform with left- or right-handed doublet indices under $SU(2)_\mathrm{L}\times SU(2)_\mathrm{R}$. These can then be contracted with the spurion operators given in Table 1 to make invariant quantities.

To be precise, a lower index on an object $x_{i_\mathrm{L}}$ leads to the transformation $x_{i_\mathrm{L}}\rightarrow \sum _{j_\mathrm{L}} (g_\mathrm{L})_{i_\mathrm{L}}^{~ j_\mathrm{L}}x_{j_\mathrm{L}}$ and equivalently for a right-handed lower index. Some examples of objects with the corresponding indices are:

10 $$\begin{aligned}&\left( Ui\tau ^2\right) _{i_\mathrm{R} j_\mathrm{L}},\quad \left( u\mathcal {N}\right) _{i_\mathrm{R}},\quad \left( u^\dagger \mathcal {N}\right) _{i_\mathrm{L}},\quad \left( u\widetilde{\mathcal {N}^c}\right) _{i_\mathrm{R}},\nonumber \\&\left( u^\dagger \widetilde{\mathcal {N}^c}\right) _{i_\mathrm{L}},\quad \left( u^\dagger u_\mu ui\tau _2\right) _{i_\mathrm{L} j_\mathrm{L}}. \end{aligned}$$

To get a neutron to antineutron transition we need an $\widetilde{\mathcal {N}^c}$ and a $\mathcal {N}$ field. Dirac (or fermion) indices are contracted between these.

The lowest order, $p^0$, operators are11and the parity-conjugates. There is no lowest order operator for $(7_\mathrm{L},1_\mathrm{R})$. The first operator that appears for $(7_\mathrm{L},1_\mathrm{R})$ is at order $p^2$:

12 $$\begin{aligned} (7_\mathrm{L},1_\mathrm{R}), p^2:\,&\left( u^\dagger \widetilde{\mathcal {N}^c}\right) _{i_\mathrm{L}} \left( u^\dagger \mathcal {N}\right) _{j_\mathrm{L}} \left( u^\dagger u_\mu ui\tau _2\right) _{k_\mathrm{L} l_\mathrm{L}}\nonumber \\&\quad \times \left( u^\dagger u_\mu ui\tau _2\right) _{m_\mathrm{L} n_\mathrm{L}} \end{aligned}$$

At higher orders there are very many operators. A partial list can be found in [18]. We will restrict ourselves to comments sufficient for the application to neutron–antineutron transitions. The relevant independent combinations we refer to as $\delta _i$ below.

At order *p*, the operators must contain a derivative $D_\mu$ or $u_\mu$. As such, they will contain either dependence on the neutron or antineutron four momentum, or contain an extra pion. For a neutron–antineutron transition at rest the HBCHPT momentum $k_\mu$ vanishes. There is thus no tree level contribution to neutron–antineutron transitions. Loop level contributions from these operators will start at $p^3$, which is beyond what is considered in this paper.

**Fig. 2:**
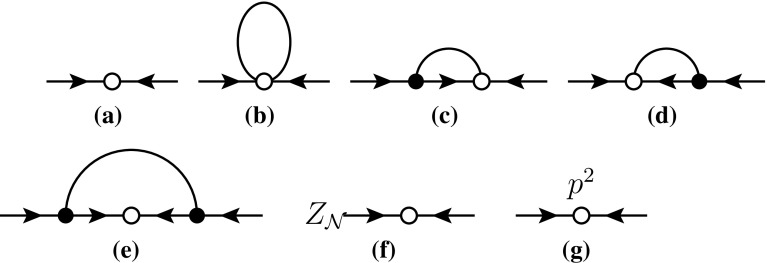
The diagrams for $n\bar{n}$ transitions to order $p^2$. An open dot indicates a vertex from the $n\bar{n}$ Lagrangian (16), a dot from the LO normal Lagrangian (7). The contributions from wave-function renormalization are indicated schematically in (f) and from the $p^2$ $n\bar{n}$-Lagrangian in (g). A right-pointing line is a neutron, a left-pointing line an antineutron

At order $p^2$ there are very many operators that contribute, a rather extensive list is in [18]. Two examples are

13 $$\begin{aligned} \left( u^\dagger D_\mu \widetilde{\mathcal {N}^c}\right) _{i_\mathrm{L}} \left( u^\dagger D^\mu \mathcal {N}\right) _{j_\mathrm{L}},\quad \left( u^\dagger \widetilde{\mathcal {N}^c}\right) _{i_\mathrm{L}} \left( \chi ^\dagger u\mathcal {N}\right) _{j_\mathrm{L}}. \end{aligned}$$

For this paper it is sufficient to notice that there is a free parameter at order $p^2$ associated with each operator.

How many parameters do we need to order $p^2$ to describe neutron–antineutron transitions given the operators $P_1,\ldots ,P_7$ with a given coefficient? The operators $P_1,P_2,P_3$ are all $(3_\mathrm{L},1_\mathrm{R})$, however the quark-operators are not related by a chiral transformation. This leads to three free parameters at order $p^0$ and three more at order $p^2$. The three operators $P_5,P_6,P_7$ belong to same chiral multiplet, i.e. they are related via a chiral transformation. This leads to one parameter at $p^0$ and one more at $p^2$. The $(7_\mathrm {L},1_\mathrm {R})$ operator at order $p^2$ does not contribute to neutron–antineutron transitions.

The values to which the spurions need to be set to reproduce the quark level operators can be derived from the expressions in [11]. They are (1 corresponds to an up-quark, 2 to a down-quark):

14 $$\begin{aligned} \theta _1^{ij}&=\theta _2^{ij} =\theta _3^{ij} = \delta ^i_2 \delta ^j_2, \nonumber \\ \theta _5^{ijklmn}&=\delta ^i_1\delta ^j_1\delta ^k_2\delta ^l_2\delta ^m_2\delta ^n_2, \nonumber \\ \theta _6^{ijklmn}&=\frac{1}{2\sqrt{2}} \left( \delta ^i_1\delta ^j_2+\delta ^i_2\delta ^j_1\right) \left( \delta ^k_1\delta ^l_2\delta ^m_2\delta ^n_2 +\delta ^k_2\delta ^l_1\delta ^m_2\delta ^n_2\right. \nonumber \\&\quad \left. +\,\delta ^k_2\delta ^l_2\delta ^m_1\delta ^n_2 +\delta ^k_2\delta ^l_2\delta ^m_2\delta ^n_1\right) , \nonumber \\ \theta _7^{ijklmn}&=\frac{1}{\sqrt{6}} \delta ^i_2\delta ^j_2 \left( \delta ^k_1\delta ^l_1\delta ^m_2\delta ^n_2 +\delta ^k_1\delta ^l_2\delta ^m_1\delta ^n_2 +\delta ^k_1\delta ^l_2\delta ^m_2\delta ^n_1\right. \nonumber \\&\quad \left. +\,\delta ^k_2\delta ^l_1\delta ^m_1\delta ^n_2 +\delta ^k_2\delta ^l_1\delta ^m_2\delta ^n_1 +\delta ^k_2\delta ^l_2\delta ^m_1\delta ^n_1\right) . \end{aligned}$$

Note that these are normalized to 1, slightly different from [11].

To summarize the neutron–antineutron part. If the Lagrangian at the quark-level is of the form

15 $$\begin{aligned} \sum _{i=1,7} \alpha _i P_i \end{aligned}$$

then the LO ChPT Lagrangian has the form

16 $$\begin{aligned} \mathcal {L}_{n\bar{n}}&= \left( \beta _1\alpha _1+\beta _2\alpha _2+\beta _3\alpha _3\right) \theta _1^{i_\mathrm{L}j_\mathrm{L}}R_{i_\mathrm{L}j_\mathrm{L}}\nonumber \\&\quad +\beta _5\left( \alpha _5\theta _5^{i_\mathrm{L}j_\mathrm{L}k_\mathrm{R}l_\mathrm{R}m_\mathrm{R}n_\mathrm{R}}+\alpha _6\theta _6^{i_\mathrm{L}j_\mathrm{L}k_\mathrm{R}l_\mathrm{R}m_\mathrm{R}n_\mathrm{R}}\right. \nonumber \\&\quad \left. +\alpha _7\theta _7^{i_\mathrm{L}j_\mathrm{L}k_\mathrm{R}l_\mathrm{R}m_\mathrm{R}n_\mathrm{R}}\right) R_{i_\mathrm{L}j_\mathrm{L}k_\mathrm{R}l_Rm_Rn_R} \end{aligned}$$

with the spurions as defined in (14) and the operators in (11). The $\alpha _i$ are short-distance parameters while the $\beta _i$ are long-distance parameters. The parity-conjugate operators can be included similarly.

**Fig. 3:**
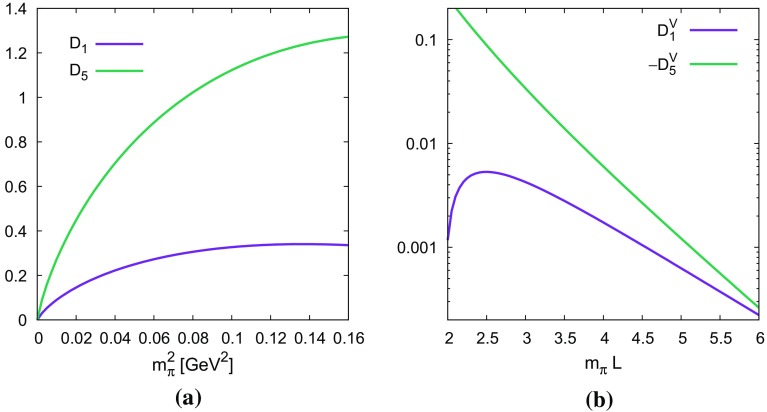
The numerical results of the pure loop contributions. **a** The infinite volume correction of (24). **b** The finite volume correction of (25)

## Analytical results

The diagrams needed for $n\bar{n}$ transition to order $p^2$ are shown in Fig. 2.

The LO, $p^0$, result from Fig. 2a is

17 $$\begin{aligned} A(n\rightarrow \bar{n})_{LO}&= \beta _1\alpha _1+\beta _2\alpha _2+\beta _3\alpha _3\nonumber \\&\quad +\beta _5\left( \alpha _5-\frac{\alpha _6}{\sqrt{2}}+\frac{\alpha _7}{\sqrt{6}}\right) . \end{aligned}$$

The integrals we use are defined in Appendix B. The tadpole diagram of Fig. 2b contributes

18 $$\begin{aligned} A(n\rightarrow \bar{n})_{(b)}&=\frac{1}{F^2} A(m_\pi ^2)\Bigg [\left( \beta _1\alpha _1+\beta _2\alpha _2+\beta _3\alpha _3\right) \nonumber \\&\quad +7\beta _5\left( \alpha _5-\frac{\alpha _6}{\sqrt{2}}+\frac{\alpha _7}{\sqrt{6}}\right) \Bigg ]. \end{aligned}$$

The diagrams (c) and (d) contain the integral

19 $$\begin{aligned} \frac{1}{i}\int \frac{d^d r}{(2\pi )^d}\frac{S\cdot r}{(r^2-m_\pi ^2)(v\cdot (r+k))}. \end{aligned}$$

We work in the frame where the external momentum *k* vanishes. In infinite volume the integral is proportional to $v\cdot S=0$. In finite volume for a neutron and antineutron at rest, *S* is purely spatial, and the integral/sum is odd under $\mathbf {r}\rightarrow -\mathbf {r}$ and vanishes for periodic boundary conditions. So (c) and (d) give no contribution.

Diagram (e) can be rewritten in terms of the integral

20 $$\begin{aligned} I(m_\pi ^2) = \frac{1}{i}\int \frac{\mathrm{d}^dr}{(2\pi )^2}\frac{\left( S\cdot r\right) ^2}{(r^2-m_\pi ^2) (v\cdot r)^2}. \end{aligned}$$

The central vertex is directly the LO contribution so (f) contributes

21 $$\begin{aligned} A(n\rightarrow \bar{n})_{(f)} =-\frac{g_A^2}{F^2}I(m_\pi ^2) A(n\rightarrow \bar{n})_{LO}. \end{aligned}$$

Wave-function renormalization can be computed from the derivative of the nucleon (and antinucleon) selfenergy. This leads again to the occurrence of the integral $I(m_\pi )^2$ in this contribution. We get

22 $$\begin{aligned} A(n\rightarrow \bar{n})_{(f)} =\frac{3g_A^2}{F^2}I(m_\pi ^2) A(n\rightarrow \bar{n})_{LO}. \end{aligned}$$

Depending on the form of $p^3$ Lagrangian in the pion nucleon sector chosen, we have a contribution proportional to $m_\pi ^2$ and a possible $p^3$ pion-nucleon LEC. This is nonzero if choosing the Lagrangian in [22] and vanishes if the version of [23] is chosen. The two choices are related by a field redefinition. The effect is that the $p^2$ $n\bar{n}$ LECs (referred to as $\delta _i$ below) have different values in the two cases but such that the total result remains the same.

The final result is

23 $$\begin{aligned} A(n\rightarrow \bar{n})&= \left( \beta _1\alpha _1+\beta _2\alpha _2+\beta _3\alpha _3\right) \left[ 1+\frac{1}{F^2}\left( A(m_\pi ^2)\right. \right. \nonumber \\&\quad \left. \left. +2g_A^2 I(m_\pi ^2)\right) \right] \nonumber \\&\quad +\beta _5\left( \alpha _5-\frac{\alpha _6}{\sqrt{2}}+\frac{\alpha _7}{\sqrt{6}}\right) \left[ 1+\frac{1}{F^2}\left( 7A(m_\pi ^2)\right. \right. \nonumber \\&\quad \left. \left. +2g_A^2 I(m_\pi ^2)\right) \right] \nonumber \\&\quad +m_\pi ^2\left( \delta _1\alpha _1+\delta _2\alpha _2+\delta _3\alpha _3\right) \nonumber \\&\quad +m_\pi ^2\delta _5\left( \alpha _5-\frac{\alpha _6}{\sqrt{2}}+\frac{\alpha _7}{\sqrt{6}}\right) . \end{aligned}$$

In order to get the infinite volume finite result, replace the $\delta _i$ by their finite parts $\delta _i^r$ and the integrals *I*, *A* by $\overline{I}, \overline{A}$. The finite volume correction is obtained by dropping terms not involving an integral and replacing *I*, *V* by $I^V,A^V$. Expressions for these integrals are in Appendix B.

## Numerical results

We set in this section all $p^2$ LECs, $\delta _i^r$, to zero.

The relative chiral correction from the loops to $$(3_\mathrm{L},1_\mathrm{R})$$ ($$D_1)$$) and $$(3_\mathrm{L},5_\mathrm{R})$$ ($$D_5$$) operators is given by keeping the *I*, *A* terms in (23) and replacing them by $$\overline{I},\overline{A}$$. The result is

24 $$\begin{aligned} D_1&= \frac{m_\pi ^2}{16\pi ^2 F^2}\left[ \left( -1-\frac{3g_A^2}{2}\right) \log \frac{m_\pi ^2}{\mu ^2}-g_A^2\right] , \nonumber \\ D_5&= \frac{m_\pi ^2}{16\pi ^2 F^2}\left[ \left( -7-\frac{3g_A^2}{2}\right) \log \frac{m_\pi ^2}{\mu ^2}-g_A^2\right] . \end{aligned}$$

These are plotted in Fig. 3a for a range of $m_\pi ^2$ with $F=92.2$ MeV fixed and $g_A=1.25$. Note that they are large for the $(3_\mathrm{L},5_\mathrm{R})$ operators already at $m_\pi \approx 200$ MeV.

The correction due to finite volume is obtained by replacing *I*, *A* by $I^V,A^V$ in (23):

25 $$\begin{aligned} D_1^V&= \frac{1}{F^2}\left[ \left( 1+\frac{g_A^2}{2}\right) A^V(m_\pi ^2,1) +m_\pi ^2 g_A^2 A^V(m_\pi ^2,2)\right] , \nonumber \\ D_5^V&= \frac{1}{F^2}\left[ \left( 7+\frac{g_A^2}{2}\right) A^V(m_\pi ^2,1) +m_\pi ^2 g_A^2 A^V(m_\pi ^2,2)\right] . \end{aligned}$$

These are plotted in Fig. 3b for $m_\pi =135$ MeV and $F=92.2$ MeV as a function of $m_\pi L$. $D_5^V$ is negative over the whole region while $D_1^V$ is positive. $D_1^V$ goes through zero just below the region plotted. The finite volume corrections are small for $m_\pi L>4$.

## Conclusions

In this paper we have constructed ChPT operators for the dimension 9 six-quark operators that contribute to neutron–antineutron oscillations. At order $p^0$ there is one term each transforming as $(3_\mathrm{L},1_\mathrm{R})$ and $(3_\mathrm{L},5_\mathrm{R})$. The $(7_\mathrm{L},1_\mathrm{R})$ operators only contribute at order $p^3$ by power-counting but do require isospin violation. We showed that the order *p* operators only contribute from order $p^3$. There is a large number of operators contributing at order $p^2$, a partially complete list can be found in [18]. The same is true for the parity-conjugate operators.

Our main results are the one-loop corrections in (23), (24) and (25). We have shown numerical results. The finite volume corrections are small for $m_\pi L > 4$. We found that chiral corrections are reasonable for the $(3_\mathrm{L},1_\mathrm{R})$ operators but can be sizable for the $(3_\mathrm{L},5_\mathrm{R})$ operators.

## Group theory

*SU*(2) is a pseudoreal group with as generators $T^a=(1/2)\tau ^a$. The Pauli matrices $\tau ^a$ are Hermitian and satisfy

26 $$\begin{aligned} \tau ^{aT} = \tau ^{a*} = -\tau ^2\tau ^a\tau ^2. \end{aligned}$$

As a consequence the special unitary matrices $x=g_\mathrm{L},g_\mathrm{R},u,h$ all satisfy

27 $$\begin{aligned} \tau ^2 x \tau ^2 =\,&x^*,&\tau ^2 x^T \tau ^2 = x^\dagger . \end{aligned}$$

These identities are used a lot in the construction of the transformations and operators in the main text.

## Integrals

The integrals we need to calculate both at infinite and finite volume. In finite volume we replace the integral over spatial momenta by a sum. The techniques are well known both at finite and infinite volume, we use here [22] and [25].

The mesonic integral/sum needed is

28 $$\begin{aligned} A(m^2)&=\frac{1}{i}\int \frac{\mathrm{d}^dr}{(2\pi )^d}\frac{1}{r^2-m_\pi ^2} = \frac{\lambda _0}{16\pi ^2}\nonumber \\&\quad +\overline{A}(m^2)+A^V(m^2,1), \end{aligned}$$

with

29 $$\begin{aligned} \lambda _0 =\,&\frac{1}{\epsilon }+1+\log (4\pi )-\gamma _E,&d=\,&4-2\epsilon . \end{aligned}$$

The terms with $\lambda _0$ are removed by the renormalization procedure and the logarithms of $m_\pi ^2$ obtain the subtraction scale $\mu ^2$ via the renormalization as well. We therefore quote the integrals including $\mu ^2$. The finite volume part depends on the spatial length scale *L*. The results are, see e.g. [25],

30 $$\begin{aligned} \overline{A}(m_\pi ^2)&=-\frac{m_\pi ^2}{16\pi ^2}\log \frac{m_\pi ^2}{\mu ^2}, \nonumber \\ A^V(m^2,n)&=\frac{(-1)^n}{16\pi ^2}\left( \frac{L^2}{4}\right) ^{n-2} \int \frac{\mathrm{d}\lambda }{\Gamma (n)}\nonumber \\&\quad \lambda ^{n-3}e^{-\lambda m^2 L^2/4} \left[ \theta _3\left( e^{-1/\lambda }\right) ^3-1\right] , \nonumber \\ \theta _3&=\sum _{n=-\infty ,\infty } q^{(n^2)}. \end{aligned}$$

The other integral/sum needed is

31 $$\begin{aligned} I(m_\pi ^2) = \frac{1}{i}\int \frac{\mathrm{d}^dr}{(2\pi )^d} \frac{\left( S\cdot r\right) ^2}{(r^2-m_\pi ^2)(v\cdot r)^2}. \end{aligned}$$

The numerator can be rewritten via

32 $$\begin{aligned} \left( S\cdot r\right) ^2&= \frac{1}{2} r^\mu r_\nu \left\{ S_\mu ,S_\nu \right\} = \frac{1}{4}\left[ (v\cdot r)^2-r^2\right] \nonumber \\&=\frac{1}{4}\left[ (v\cdot r)^2-(r^2-m_\pi ^2)-m_\pi ^2\right] . \end{aligned}$$

The second term leads to an integral with only $v\cdot r$ in the denominator. These vanish both at infinite and finite volume. We thus get

33 $$\begin{aligned} I(m_\pi ^2) = \frac{1}{4}A(m_\pi ^2)-\frac{m_\pi ^2}{4} \frac{1}{i}\int \frac{\mathrm{d}^dr}{(2\pi )^d} \frac{1}{(r^2-m_\pi ^2)(v\cdot r)^2} \end{aligned}$$

We combine the propagators in the second term with $1/(ab^2)=\int _0^\infty \mathrm{d}\lambda \, 8\lambda /(a+2b\lambda )^3$ and shift the momentum to^2^ $\tilde{r}=r+v\lambda$ and obtain the integral

34 $$\begin{aligned}&\frac{1}{i}\int \frac{\mathrm{d}^d\tilde{r}}{(2\pi )^d} \int _0^\infty \mathrm{d}\lambda 8\lambda \frac{1}{\left( r^2-(m_\pi ^2+\lambda ^2)\right) ^3} \nonumber \\&\quad = -\frac{1}{i}\int \frac{\mathrm{d}^d\tilde{r}}{(2\pi )^d}\frac{2}{\left( \tilde{r}^2-m_\pi ^2\right) ^2}. \end{aligned}$$

In the second step we have done the $\lambda$-integral. The integral/sum appearing now is known and we obtain

35 $$\begin{aligned} I(m_\pi ^2)&= \frac{\lambda _0}{16\pi ^2}\frac{3m_\pi ^2}{4}+\overline{I}(m_\pi ^2) +I^V(m_\pi ^2), \nonumber \\ \overline{I}(m_\pi ^2)&= \frac{m_\pi ^2}{16\pi ^2}\left( -\frac{3}{4}\log \frac{m_\pi ^2}{\mu ^2}-\frac{1}{2}\right) , \nonumber \\ I^V(m_\pi ^2)&=\frac{1}{4}A^V(m_\pi ^2,1)+\frac{m_\pi ^2}{2}A^V(m_\pi ^2,2). \end{aligned}$$

## Footnotes

1 In this equation, the fields $p^c,n^c$ are the HBCHPT ones for the antinucleons, not the relativistic fields.
